# The prevalence of chronic kidney disease among type 2 diabetes mellitus patients in central South Africa

**DOI:** 10.4102/safp.v65i1.5663

**Published:** 2023-04-23

**Authors:** William Mhundwa, Gina Joubert, Thabiso R.P. Mofokeng

**Affiliations:** 1 Department of Internal Medicine, School of Clinical Medicine, Faculty of Health Sciences, University of the Free State, Bloemfontein, South Africa; 2 Department of Biostatistics, School of Biomedical Sciences, Faculty of Health Sciences, University of the Free State, Bloemfontein, South Africa

**Keywords:** chronic kidney disease, type 2 diabetes mellitus, end-stage renal disease, diabetic kidney disease, risk factors, control, albuminuria, renin-angiotensin-aldosterone system

## Abstract

**Background:**

Type 2 diabetes mellitus (T2DM) is a leading cause of chronic kidney disease (CKD). The prevalence of CKD among T2DM patients in Africa is 22.0%. The cut-off age for dialysing diabetic patients in the resource-limited state sector in South Africa is 50 years. Type 2 diabetes mellitus patients who develop CKD are likely to be excluded from chronic dialysis and rely on control of risk factors, including blood pressure and blood glucose levels, to prevent CKD progression. We aimed to determine the prevalence of CKD among T2DM patients attending the diabetes clinic at Pelonomi Academic Hospital, Bloemfontein.

**Methods:**

In this retrospective cross-sectional study, medical records of patients (January 2016 and December 2018) were reviewed to collect demographic and clinical information.

**Results:**

In total, 244 records were reviewed. Sixty-one (25.0%, 95% confidence interval [CI]: 20% – 30.8%) T2DM patients had CKD. The rate of CKD was slightly higher in males (*n* = 24/81; 29.6%) compared with females (*n* = 37/163; 22.7%). Most patients with CKD (*n* = 58; 95.1%) were > 50 years of age. Only 17.8% of patients achieved a glycosylated haemoglobin (HbA1c) of < 7.0%. Blood pressure was controlled in 14.3% of hypertensive patients. Renin–angiotensin–aldosterone system inhibitors were used by 78.6% of patients.

**Conclusion:**

A high prevalence of clinically significant CKD among T2DM patients with poor prospects of chronic dialysis in a resource-limited setting was observed. The risk factors for CKD development and progression should be adequately managed in T2DM patients.

**Contribution:**

This study emphasises the need for further research and innovation to improve outcomes of T2DM patients with CKD in resource-constrained settings.

## Background

Chronic kidney disease (CKD) and type 2 diabetes mellitus (T2DM) are two interdependent non-communicable diseases of which the worldwide prevalence in the general population has reached epidemic proportions.^1^ Chronic kidney disease occurs in 10%^2^ and diabetes mellitus (DM) in 9.3%^3^ of the world population. Type 2 DM represents 90% of all diabetic cases worldwide.^4^ Clinically, overt CKD has an estimated glomerular filtration rate (eGFR) persistently less than 60 mL/min over a period of at least 3 months.^5^ The worldwide prevalence of CKD among DM patients reportedly ranges between 20% and 30%.^6^ In African countries, wide variation in the prevalence of CKD has been reported, ranging between 2% and 41% of the general population, with a pooled prevalence of 10.1%.^7^

In a global study on CKD spanning a 27-year period, T2DM was the only cause of CKD showing a significant increase of 9.5% in disability-adjusted life years (DALY) rate.^8^ In South Africa, the prevalence of CKD has increased by 10.5% between 1990 and 2017, while CKD-related deaths increased by 28.8% during this period.^7,8^ Using albuminuria as a marker of diabetic nephropathy, Ngassa Piotie et al. demonstrated a prevalence of 33.6%.^9^ A Cape Town-based study showed a prevalence of stage 3–5 CKD in 23.9% of 1202 participants of mixed ancestry. Concomitant DM was identified in 26.4% of this cohort.^10^ Saeedi et al. reported a 24.7% pooled prevalence of CKD among patients with diabetes in Africa, ranging from 11% to 90% between different countries.^3^

The development of CKD in DM patients intensifies their morbidity and increases mortality, particularly from cardiovascular-related deaths. A combination of these disease entities has a major psychological and socioeconomic impact on the patients and increases the burden on the healthcare system. Chronic kidney disease needs to be prevented or diagnosed early and treatment optimised for DM, CKD and other comorbidities in order to avoid disease progression to end-stage kidney disease (ESKD).^11^ According to the American Diabetic Association (ADA), glycaemic control targeting a glycated haemoglobin (HbA1c) of less than 7% is essential to avoid the progression of CKD to dialysis-requiring ESKD.^12,13,14^ Despite increasing therapeutic options, control of diabetes remains elusive for most diabetic patients in South Africa. Behaviour modification therapy is effective in managing lifestyle diseases such as DM, yet it is under-utilised.^15^

In our poorly resourced public health setting, metformin and sulphonylureas are first-line oral antidiabetic (OAD) drugs in managing T2DM. These drugs need to be terminated when the eGFR falls below 30 mL/min. Furthermore, sulphonylureas should be administered with caution and might even be contraindicated in patients with CKD.^16,17^ In the public setting, insulin is the next option used to achieve glycaemic targets. However, insulin metabolism is impaired in CKD, resulting in a predisposition to develop hypoglycaemia that may be fatal when severe.^13,18,19^

Sodium glucose transporter 2 inhibitors (SGLT2i) and glucagon-like peptide-1 (GLP-1) have been found to promote robust HbA1c control. These drugs also reduce the progression of CKD.^20,21,22^ There is need for advocacy to make these newer drugs available for patients who are unlikely to receive chronic dialysis. Type 2 DM patients commonly have hypertension.^2^ Control of blood pressure (BP) is recommended to the guideline target of 130/80 mmHg, which is essential to diminish the progression of CKD to ESKD.^23^ Antihypertensive agents preferred to achieve this target include the renin-angiotensin-aldosterone system inhibitors (RAASi). These drugs have an anti-albuminuric effect that reduces the rate of progression of CKD.^24^ However, their use can be impeded by hyperkalaemia that may develop as a RAASi side-effect or because of poor potassium excretion that occurs in CKD.^25^

For survival, patients with ESKD require renal replacement therapy (RRT) in the form of peritoneal dialysis (PD) and haemodialysis (HD), which are bridging therapies to kidney transplants. In sub-Saharan Africa, CKD prevention is our best defence because RRT is not easily accessible due to its cost, lack of dialysis facilities and a shortage of donor organs. The annual cost of dialysis was at least R212 286.00 (South African rand) for HD and R255 076.00 for PD, which retrospectively equated to $25 888.00 (United States dollar) and $31 106.00 in 2019.^26^

Diabetic patients older than 50 years of age are excluded from RRT programmes in most South African state health centres because of resource limitations.^27^ Therefore, prevention of CKD development is the primary key to survival for diabetic patients in the public setting. All healthcare workers (HCWs) must screen patients for CKD to ensure optimal control of the risk factors associated with CKD in DM patients.

The primary aim of the study was to determine the prevalence of CKD among T2DM patients attending the diabetes clinic at Pelonomi Academic Hospital (PAH) during the period starting 01 January 2016 until 31 December 2018. The secondary objectives were to determine the percentage of patients who were reaching the recommended targets of BP and HbA1c and to compare CKD participants and those without CKD regarding the presence of some of the risk factors associated with disease progression in CKD. Our results intend to emphasise the local prevention strategies regarding DM, hypertensive nephropathy (HTN) and CKD, and ultimately reduce needless morbidity and mortality resulting from these highly treatable conditions.

## Methods

### Study design, population and setting

A retrospective cross-sectional study was conducted by reviewing files of all patients with T2DM who attended the outpatient diabetes clinic at PAH from the period 01 January 2016 to 31 December 2018. The PAH is a tertiary-level hospital situated in Bloemfontein in the Free State province of South Africa. This hospital provides healthcare to a population in both urban and rural areas of the province located in the central region of South Africa. The diabetes clinic is conducted every Thursday. The patients attending this clinic have been referred from a primary healthcare facility for specialist services because of poorly controlled blood sugar, comorbidities and complications of their DM.

### Study participants

All the patients seen during the 3-year period were recruited into the study without any sampling. It was anticipated that approximately 400 patients would qualify for the study. The following inclusion and exclusion criteria were applied.

Inclusion criteria:

- age of 18 years and above
- type 2 diabetes mellitus
- visiting the diabetes clinic during the study period

Exclusion criteria:

- patients whose files could not be traced

### Data collection

The principal researcher collected information from the patient registers that are kept at the clinic. The patients’ physical files kept in the clinic were perused to obtain their demographic and clinical information. A search for additional clinical information was performed on the MEDITECH electronic patient file system. Laboratory results were checked on the National Health Laboratory Service (NHLS) electronic database. The MEDITECH and NHLS electronic systems are password-protected electronic databases. These databases were accessed by the principal researcher using login details that are used during the usual daily duties as provided by the Department of Health (DoH).

A total of 370 patients appeared in the registers and were assigned study numbers from 1 to 370 according to their order of appearance in the clinic registers. Information was recorded on a data form for up to four different clinic visits that were at least 3 months apart. The demographic information that was collected included patient’s gender and age.

**FIGURE 1:**
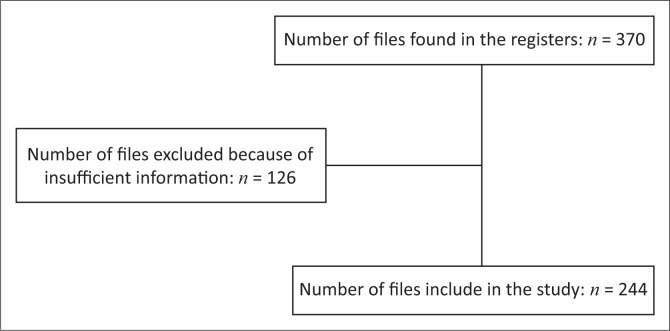
Selection of files included in the study.

A total of 126 patient files were excluded because they did not meet the inclusion criteria. Finally, 244 patient files were included in the study. Figure 1 illustrates the outcome of the file selection process.

#### Clinical information

Blood pressure was measured at each visit, and we recorded if the participant was known to have hypertension or not. The average of both systolic and diastolic values for all the visits was then calculated. When the average was found to be ≤ 130/80 mmHg, it was noticed as controlled and an average of > 130/80 mmHg as uncontrolled BP.

From the medication history, it was recorded if the patient was taking a RAASi or not. Other illnesses besides hypertension were recorded, including HIV status, heart failure, osteoarthritis, malignancy and chronic respiratory conditions.

#### Laboratory results

Laboratory results obtained at each clinic visit were entered into the data tool. These included serum creatinine levels and the corresponding eGFR as supplied by the laboratory using the Modification of Diet in Renal Disease (MDRD) equation. When the eGFR was ≤ 60 mL/min per 1.73 m^2^, the absolute value of eGFR was recorded; otherwise it was stated as > 60 mL/min per 1.73 m^2^. Where the eGFR was < 60 mL/min per 1.73 m^2^ on consecutive occasions separated by a period of at least 3 months, CKD was reported to be present. Chronic kidney disease was also recorded to be present if the patient had a diagnosis of CKD made before 01 January 2016.

Glycated haemoglobin (HbA1c) was recorded at each visit. An average HbA1c for all the visits was calculated and recorded. If the average HbA1c was ≤ 7%, it was noted as controlled and uncontrolled if > 7%. Serum potassium (K^+^) levels were recorded at each visit. A level of ≥ 5.5 mmol/L at any visit was noted to represent hyperkalaemia.

According to the study protocol, the patient’s weight, duration of living with DM, serum cholesterol level, evidence of proteinuria and renal sonar findings were to be collected. However, information on these variables was not available in most of the patient files.

The information captured for each patient on the data collection tool was then transferred to a Microsoft Excel (version 2016) spreadsheet for statistical analysis.

### Statistical analysis

Data were analysed by the department of Biostatistics of the University of the Free State using SAS version 9.4 (SAS Institution Inc., Cary, North Carolina, United States). Categorical data were summarised by frequencies and percentages. Numerical variables were summarised by medians with interquartile ranges (IQR). Denominators available for variables were indicated throughout as missing information occurred.

Subgroups were compared regarding numerical variables using with the Mann-Whitney test and regarding categorical variables using the chi-squared or Fisher’s exact test as appropriate. The level of statistical significance was set at *p* < 0.05, and 95% confidence intervals (95% CI) were calculated for main outcomes.

### Pilot study

A pilot study was carried out using the first five files. It was observed that some variables in the study protocol, such as duration of T2DM, evidence of proteinuria and cholesterol, had frequently missing information. These variables were then excluded from the main study. The pilot study cases were included in the main analysis.

### Ethical considerations

Ethical approval was obtained from the Health Sciences Research Ethics Committee (HSREC) of the University of the Free State (reference number UFS-HSD2019/2204/2502) before data collection. Permission was also granted by the Free State province Department of Health (reference number FS 201911_020). Because of the retrospective nature of the study and using archived patient files to collect data, no informed consent was required. The data captured and analysed were anonymised and no identifiable patient information was collected.

## Results

A total of 244 patients were included in this study. Table 1 summarises results for the group as well as for patients with CKD and those without CKD. The median age of patients was 62.5 years (range 29–96 years). The majority (*n* = 163; 66.8%) of patients were female. The prevalence of CKD in this study population was 25.0% (*n* = 61) (95% CI: 20% – 30.8%).

**TABLE 1:** The presence of risk factors for renal disease progression among type 2 diabetes mellitus patients and those with and without chronic kidney disease.

Variable	Total group					CKD present					No CKD					*p*
	*n*	*N*	%	Median	IQR	*n*	*N*	%	Median	IQR	*n*	*N*	%	Median	IQR	
**Gender**																
Female	163	244	66.8	-	-	37	61	60.7	-	-	126	183	68.9	-	-	0.24
Male	81	244	33.2	-	-	24	61	39.7	-	-	57	183	31.2	-	-	
Median age (years)	-	-	-	62.5	56–72	-	-	-	66	60–76	-	-	-	61	54–71	< 0.01
Hypertension	147	161	91.3	-	-	35	39	89.7	-	-	112	122	91.8	-	-	0.75
If hypertensive, blood pressure controlled (≤ 130/80 mmHg)	20	140	14.3	-	-	3	34	8.8	-	-	17	106	16.0	-	-	0.40
Blood glucose controlled (HbA1c ≤ 7%)	41	240	17.1	-	-	10	61	16.4	-	-	31	179	17.3	-	-	0.87
Comorbidities	32	156	20.5	-	-	10	41	24.4	-	-	22	115	19.1	-	-	0.47
Receiving RAASi therapy	125	159	78.6	-	-	25	39	64.1	-	-	100	120	83.3	-	-	< 0.01
Hyperkalaemia	45	242	18.6	-	-	24	59	40.7	-	-	21	183	11.5	-	-	< 0.01

CKD, chronic kidney disease; IQR, interquartile range; HbA1c, glycated haemoglobin; RAASi, renin-angiotensin-aldosterone system inhibitors.

The cases with CKD had a median age of 66 years (IQR: 60–76 years). Patients with CKD were significantly older than those without CKD, who had a median age of 61 years (IQR: 54–71 years), with *p* < 0.01 and a 95% CI of 2–9 for the median difference. Nearly all (*n* = 58/61; 95.1%) of the patients with CKD were older than the 50 years cut-off for dialysis in our setting. In terms of gender, the prevalence of CKD among male patients (*n* = 24/81; 29.6%) was higher than in females (*n* = 37/163; 22.7%). However, this difference was not significant (*p* = 0.24).

In 240 patient files, HbA1c was recorded, the majority of whom (*n* = 199; 82.9%) had HbA1c values of > 7%. Among patients with CKD, 16.4% (*n* = 10/61) had controlled HbA1c, compared with 17.3% of patients without CKD (*n* = 31/179, *p* = 0.87).

Hypertensive status was recorded in 161 patient files. In this diabetic cohort, most patients (*n* = 147/161; 91.3%) were hypertensive, while 35/147 (23.8%) of patients with hypertension had CKD. The proportion of hypertensive T2DM patients with controlled BP was only 14.3% (*n* = 20/140), while 85.7% (*n* = 120/140) failed to reach the recommended BP target of 130/80 mmHg. The majority of hypertensive patients with CKD (*n* = 31/34; 91.2%) had uncontrolled BP. Of all the patients, 17.5% (*n* = 27/154) had controlled BP, while 13.2% of patients with CKD and 19.0% (*n* = 22/116) of patients without CKD had controlled BP.

In 159 files, information regarding RAASi was recorded, with 78.6% (*n* = 125/159) of these patients receiving RAASi. In 7 (4.4%) patients on RAASi, treatment had been terminated because of either drug reactions or hyperkalaemia. These seven patients all had CKD as well. A significant association (*p* < 0.01) was observed regarding treatment with RAASi and CKD. Slightly more than 60% (*n* = 25/39; 64.1%) of patients with CKD were taking RAASi, compared with 83.3% (*n* = 100/120) of patients without CKD.

Serum potassium levels were recorded in 242 patient files, of whom 45 (18.6%) had elevated levels exceeding 5.5 mmol/L. Among 59 patients with CKD, 24 (40.7%) had hyperkalaemia, compared with 21 of 183 (11.5%) patients without CKD (*p* < 0.01). Four out of 24 (16.7%) of CKD patients on RAASi therapy had hyperkalaemia. For those who had no CKD, 9 out of 100 (9.0%) patients presented with hyperkalaemia while on RAASi.

It was possible to determine whether the patient had another illness or not in 156 files. Of these, 32 (20.5%) patients had other illnesses. Chronic kidney disease was present in 41 (26.3%) of the 156 patients with comorbidities. In this group, 10/41 (24.4%) patients with CKD had other illnesses compared with 22/115 (19.1%) patients without CKD who had other illnesses (*p* = 0.47).

## Discussion

The results of this study revealed that 25.0% of T2DM patients attending the diabetes clinic at Pelonomi Academic Hospital had concomitant CKD. This finding was notably higher than the 12.4% prevalence of CKD among diabetic patients in Tanzania,^28^ slightly lower than the prevalence of 29.6% in a Chinese study,^29^ but substantially lower than a prevalence of 83.7% reported from another Tanzanian study.^30^

Even though women with T2DM are more prone to develop complications,^31^ the prevalence of CKD among diabetic males was slightly higher (39.7%) compared with female patients (31.2%) in our study.

In this study, 95.1% of our diabetic patients who had CKD (*n* = 61) were older than 50 years, the cut-off age for life-saving dialysis according to our institutional guidelines. The patients seen in our public setting depend on social grants and, therefore, may not afford dialysis in a private healthcare facility.

Regarding HbA1c control, only 16.3% of our TDM with CKD patients met the target, while 17.4% without CKD were meeting the target. Ngassa Piotie et al. demonstrated a similar outcome in their 2015 Pretoria-based study where 88.9% of their patients had HbA1c values exceeding 7%.^9^

Most (91.3%) of the patients attending our clinic had hypertension. Only 13.2% of those with CKD were achieving recommended BP targets. This resonates well with a study carried out by Webb et al. in Tshwane, where T2DM patients were found to have an elevated mean systolic BP of 143 mmHg.^32^

However, it was encouraging that many of the patients were on RAASi, 64.1% of those with CKD and 83.3% without CKD. Ngassa Piotie et al. showed that 70% of the patients in their study were on an angiotensin converting enzyme (ACE)inhibitor.^9^ The RAASi appeared to be well tolerated, because only a minority (18.6%) of patients on RAASi developed hyperkalaemia, even among the CKD group. Studies have shown reduced CKD progression with RAASi treatment because of their antiproteinuric and BP control effect.^33,34^ The antiproteinuric effect of RAASi was not ascertained in our study because urinalysis had either not been performed or recorded in most of the patients.

### Study limitations

A limitation of this study is that the diabetic patients who are managed at a tertiary hospital by specialist physicians are likely to have complications of DM and therefore are at high risk of CKD.

Inconsistencies in the recording of information from the files were a general problem, with a substantial number of files lacking some vital information such as weight, duration of DM, BP recordings and medication prescribed. These shortcomings could be attributed to the high turnover of clinicians managing the patients visiting the diabetes clinic because of interdepartmental staff rotation. This does not only affect outcomes of the study but also compromises patient care.

The definition of CKD that was used in this study was an eGFR persistently less than 60 mL/min per 1.73 m^2^ over a period of at least 3 months. However, this parameter is applicable to patients with stage 3–5 CKD and may underestimate the actual prevalence of CKD if urine albumin to creatinine ratio (ACR) is used. The ACR could not be used in this study because of the unavailability of data in the patient records.

### Recommendations

The following are recommendations from the study:

- Clinician factors: To improve control of risk factors by using guideline-recommended therapies to achieve targets.
- Patient factors: Must be educated on their disease and need for behaviour modification.
- System factors: Increase public awareness on DM, CKD, hypertension, provision of better medications such as SGLT2i and train staff at primary care level on the latest guidelines.

## Conclusion

The authors report a high (25.0%) prevalence of clinically significant CKD among T2DM patients in a resource-limited setting. Many of the patients with CKD were above the state-required cut-off age for dialysis therapy. Control of the risk factors for CKD development and progression in our public setting is poor and needs to be improved.

In the public sector with limited resources, HCWs seeing patients with concomitant T2DM and CKD must collaborate with the patients to control risk factors for CKD development and progression and counsel the patients on the clinical implications of uncontrolled BP and glucose levels. Lifestyle modification must be encouraged. Younger patients in particular should be made aware of the cut-off age of 50 years for dialysis in the resource-restricted public healthcare setting, and the consequences should be clearly emphasised. Policymakers must be encouraged to devise a framework that enables optimum care for T2DM through the promotion of behavioural modification and the provision of effective newer drugs at affordable prices. The arbitrary cut-off age of 50 years for dialysis needs to be revised upwards.
